# Supplementary material for: WordCommentsAnalyzer: A windows software tool for qualitative research
Source: F1000Res. 2018 Sep 4;7:536. Originally published 2018 May 3. [Version 2] doi: 10.12688/f1000research.14819.2 (PMC6137411; doi:10.12688/f1000research.14819.2)
Supplement: Supplementary file 3 [file f1000research-7-17614-s0002.tgz › b2a79496-4ada-47c4-9682-a99ce867836d.zip!/Eight Iranian Health Journals/The Journal of Toloo-e-Behdasht_1.docx]

© The Journal of Toloo-e-Behdasht. This work is licensed under CC BY 4.0 (https://creativecommons.org/licenses/by/4.0/).

Volume & Issue: دوره 17 - شماره 1

Title: تعیین میزان تاثیر آموزش والدین بر مبنای نظریه شناختی- اجتماعی برکاهش مصرف میان وعده های ناسالم در دانش آموزان دختر پایه اول و دوم ابتدایی شهر اقلید

http://tbj.ssu.ac.ir/article-1-1651-fa.html

مقدمه: امروزه  مصرف زیاد تنقلات و میان وعده های ناسالم در دانش آموزان بسیار شایع است. مطالعه حاضر  با هدف بررسی تاثیر آموزش والدین بر کاهش مصرف میان وعده های ناسالم در دانش آموزان دختر پایه اول و دوم ابتدایی شهر اقلید بر اساس نظریه شناختی اجتماعی انجام شد. روش بررسی: این مطالعه از نوع مداخله ای بوده و تعداد 102 نفر دانش آموز پایه اول و دوم    (51 نفر در گروه مداخله و 51 نفر در گروه کنترل) در آن شرکت داشتند. مداخله در چارچوب نظریه شناختی- اجتماعی که شامل سازه های ....صورت گرفته و طی دو جلسه آموزشی برای والدین با روش بحث گروهی و پرسش و پاسخ و توزیع جزوه آموزشی صورت گرفت. ابزار گردآوری اطلاعات، پرسشنامه بوده که به منظور ارزیابی تاثیر مداخله، در دو مرحله قبل و سه ماه بعد از مداخله توسط والدین دانش آموزان تکمیل گردید. یافته ها: پس از مداخله، میانگین  نمره بسامد مصرف میان وعده های ناسالم مورد بررسی در دانش آموزان گروه مداخله از76/7±   5/16 در قبل از مداخله به 29/5±  39/12 پس از مداخله رسید که کاهش معنی داری را نشان می دهد ( 0001/0P= )   اما در گروه کنترل تغییر معنی داری در مصرف این مواد غذایی مشاهده نشد (352/0p=). از میان سازه  های نظریه شناختی- اجتماعی نیز برای آگاهی  ( 002/0p= )،  نگرش نسبت به پیامد رفتار(0001/0p= )،  دسترسی ( 001/0p= )، و حمایت اجتماعی      ( 0001/0p= )  افزایش معنی داری را نشان داد. نتیجه گیری: با توجه به نتایج بدست آمده می توان گفت که یک مداخله خانواده محور بر اساس آموزش والدین در چارچوب نظریه شناختی- اجتماعی می تواند در کاهش مصرف میان وعده های ناسالم در دانش آموزان دبستانی موثر باشد.

Title: مقایسه مدل درخت تصمیم و رگرسیون لوجستیک در ارزیابی پوکی استخوان

http://tbj.ssu.ac.ir/article-1-1209-fa.html

مقدمه: تشخیص زودهنگام استئوپروز کلیدی برای پیشگیری است، لیکن تشخیص، بدون استفاده از روش های تشخیصی مناسب ، به علت پیچیدگی عوامل خطر استئوپروز و روند تدریجی از دست رفتن استخوان مشکل است هدف این مطالعه ارائه و سنجش کارایی یک الگوی پیشگویی استئوپروز با استفاده از تکنیک درخت تصمیم به عنوان یک روش تشخیصی  براساس عوامل خطر در دسترس است تا  به وسیله آن افراد درمعرض خطر  برای انجام فعالیت های پیشگیرانه  شناسایی شوند. روش بررسی : برای انجام این مطالعه از داده های  131 زن با سن 20 تا 40 سال  استفاده شد. متغیر پاسخ  مقدار تراکم استخوان (t-score  ) ناحیه 4L-1L  کمری بود که به دوگروه نرمال(1- =< t-score) و در معرض خطر استئوپروز (1- >  t-score) تقسیم شد. به منظور تعیین عوامل خطر استئوپروز از مدل درخت تصمیم به روش ارزیابی متقاطع با  4=k  و رگرسیون لوجستیک استفاده شد. برای بررسی دقت پیش بینی دو مدل،  سطح زیرمنحنی مشخصه عملکرد  (AUROC)به کار گرفته شد. تحلیل داده ها با نرم افزار R انجام شد. یافته ها: سه متغیر تعداد حاملگی ، BMI و میزان کلسیم به عنوان عوامل خطر استئوپروز از مدل درختی به دست آمد و سطح زیر منحنی مشخصه عملکرد  درخت تصمیم و رگرسیون لوجستیک به ترتیب برابر  665/0  و 686/0  حاصل شد. نتیجه گیری:  سطح زیر منحنی مشخصه عملکرد برتری نسبی رگرسیون لوجستیک را نشان داد که با توجه به مزایای درخت تصمیم به کار گیری همزمان دو روش پیشنهاد می شود.

Title: آگاهی، نگرش و عملکرد رانندگان شهر یزد در زمینه راهکارهای کاهش مصرف سوخت و کنترل آلودگی هوای ناشی از خودرو

http://tbj.ssu.ac.ir/article-1-2231-fa.html

مقدمه: وسایل نقلیه موتوری از منابع مهم مصنوعی آلودگی هوا محسوب می­شوند و در این میان، اتومبیل­های شخصی سهم 90 درصدی از کل انرژی مصرف شده در بخش حمل و نقل را دارند. عوامل انسانی نقش بسیار مهمی بر میزان آلودگی هوای ناشی از خودرو بر عهده دارند. لذا مطالعه حاضر با هدف بررسی آگاهی، نگرش و عملکرد رانندگان شهر یزد در زمینه راهکارهای کاهش مصرف سوخت و کنترل آلودگی هوای ناشی از خودرو انجام شد. روش بررسی: این مطالعه توصیفی- تحلیلی بصورت مقطعی انجام شد و جامعه مورد بررسی، رانندگان  شهر یزد می­باشند. ابزار جمع آوری داده ها، یک پرسشنامه محقق ساخته بود که شامل متغیرهای جمعیتی،آگاهی، نگرش و عملکرد بود. نمونه­گیری به روش تصادفی ساده بود و در مجموع 250 پرسشنامه تکمیل شد. اطلاعات بدست آمده پس از واردن کردن در نرم افزار آماری SPSS، مورد تجزیه و تحلیل قرار گرفتند. یافته­ ها: نتایج نشان داد که با افزایش سن، میزان آگاهی، نگرش و عملکرد رانندگان بطور معناداری افزایش می­یابد (05/0 >P). مطابق با نتایج، میانگین نمره آگاهی، نگرش و عملکرد رانندگان مرد در زمینه راهکارهای کاهش مصرف سوخت و کنترل آلودگی هوای ناشی از خودرو بیشتر از رانندگان زن می باشد. میزان آگاهی افرادی که سابقه رانندگی کمتر از 2 سال داشتند، به طور معناداری کمتر بود  (000/0 =P) و نگرش رانندگانی که سابقه بیش از 5 سال داشتند بصورت معناداری بیشتر بود (002/0 =P). آگاهی 186 نفر (4/74%) از رانندگان در مورد راهکارهای کاهش مصرف سوخت و کنترل آلودگی هوای ناشی از خودرو، متوسط و عملکرد 161 نفر (4/64%) از آنان در حد متوسط بود. از نظر آماری بین متغیرهای آگاهی، نگرش و عملکرد رانندگان، همبستگی مثبت وجود داشت. نتیجه­ گیری:آگاهی، نگرش و عملکرد رانندگان شهر یزد در زمینه راهکارهای کاهش مصرف سوخت و کنترل آلودگی هوای ناشی از خودرو، متوسط می باشد و این افراد نیاز به آموزش بیشتری دارند.

Title: تأثیر مداخله رفتار درمانی دیالکتیکی بر نگرش به زندگی زنان افسرده

http://tbj.ssu.ac.ir/article-1-2387-fa.html

مقدمه: افسردگی یکی از شایعترین اختلالات روانپزشکی است که نگرش به زندگی را در افراد تحت تأثیر قرار می دهد. هدف از این پژوهش بررسی اثر بخشی رفتاردرمانی دیالکتیک بر نگرش به زندگی زنان افسرده بود. روش بررسی: این پژوهش از نوع مطالعات شبه آزمایشی با پیش آزمون-پس آزمون و گروه کنترل بود که در 30 زن افسرده مراجعه کننده به مرکز اورژانس اجتماعی شهرستان میبد انجام شد. شرکت کنندگان با استفاده از نمونه گیری هدفمند از جامعه انتخاب و به صورت تصادفی در دو گروه آزمایش و کنترل (هر گروه 15 نفر) جایگزین شدند. ابتدا شرکت کنندگان هر دو گروه به پیش آزمون نگرش به زندگی پاسخ دادند. سپس برنامه مداخله ای برای آزمودنی‏های گروه آزمایش در 9 جلسه 2 ساعته برگزار شد، درحالی که به شرکت کنندگان گروه کنترل هیچ آموزشی ارائه نشد. بعد از اتمام آزمایش و دو ماه پس از آن، از هر دو گروه آزمایش و کنترل، پس آزمون و آزمون پیگیری به عمل آمد. درنهایت داده های جمع آوری شده با استفاده از آزمون تحلیل کوواریانس یک راهه (آنکووا) مورد تجزیه و تحلیل قرار گرفتند. یافته ها: نتایج نشان داد که نمرات شرکت کنندگان گروه آزمایش در پرسشنامه نگرش به زندگی و زیرمقیاس های آن (هدف در زندگی، انتخاب/مسئولیت، پیوستگی، پذیرش مرگ، خلاء وجودی و جستجوی هدف) به طور معنی داری نسبت به گروه کنترل در پس آزمون افزایش پیدا کرده و این افزایش تا مرحله پیگیری حفظ شده بود (01/0>p). نتیجه گیری: نتایج این مطالعه نشان می دهد که رفتار درمانی دیالکتیک می تواند روش مناسبی جهت اصلاح نگرش به زندگی در زنان افسرده باشد.

Title: بررسی پیشگویی کننده های وضعیت بهداشت دهان ودندان در مادران باردار شهریزد بر اساس تئوری رفتار برنامه ریزی شده

http://tbj.ssu.ac.ir/article-1-2704-fa.html

مقدمه :با توجه به شرایط خاص فیزیولوژیک و آسیب پذیری  زنان باردار لازم است تاکید بیشتری بر آموزش و رعایت اصول بهداشت دهان ودندان در این دوران صورت پذیرد از آنجا که اکثر مشکلات بهداشتی با رفتار انسان ارتباطی تنگا تنگ دارند تئوریهای مطالعه رفتار می توانند برای درک چگونگی پیشگیری از مشکلات بهداشتی دهان ودندان مورد استفاده قرار گیرند. از این روهدف از این مطالعه تعیین پیشگویی کننده های وضعیت  بهداشت دهان ودندان مادران باردار بر اساس تئوری رفتار برنامه ریزی شده  بود. روش بررسی: در این مطالعه توصیفی ازنوع همبستگی تعداد 208 نفر از مادران باردار مراجعه کننده به 4 مرکز بهداشتی درمانی شهر یزد به روش نمونه گیری تصادفی چند مرحله ای انتخاب شدند واز طریق مصاحبه  پرسشنامه ها تکمیل شد. (روایی پرسشنامه توسط نظرات متخصصان وپایایی بامحاسبه آلفای کرونباخ 83/0-64/0  تأیید شده بود این پرسشنامه بر اساس تئوری رفتار برنامه ریزی شده طراحی شد و شامل اطلاعات دموگرافیک وزمینه ای وسوالات سازه های تئوری  و وضعیت بهداشت دهان ودندان خود گزارش شده بود. یافته ها: ازمادران باردار مورد بررسی 1/59 درصد یکبار در روز مسواک می‌زدند وتنها 8/30درصد  یکبار در روز از نخ دندان استفاده می کردند و میانگین تعداد دندانهای سالم مادران برابر با 65/7 ± 56/23 بود ازبین سازه های مدل تئوری رفتار برنامه ریزی شده سازه های نگرش، هنجار ذهنی وکنترل رفتاری درک شده 66% قصد رفتاری را پیشگویی کردند ورفتار بهداشتی 12% تغییرات وضعیت بهداشت دهان ودندان را پیشگویی می کرد از بین سازه های مدل ،کنترل رفتاری درک شده قوی ترین پیشگویی کننده قصد رفتاری بود. نتیجه گیری : بکار گیری تئوری رفتار برنامه ریزی شده می تواند با تمرکز بر روی کنترل رفتاری درک شده نتایج ثمر بخشی را در زمینه رفتار مطلوب بهداشت دهان ودندان به بار آورد این مدل می تواند بعنوان یک مدل  مناسب در برنامه های ارتقاء  سلامت دهان ودندان بکار رود.

Title: بررسی تاثیر بازی درمانی با رویکرد شناختی رفتاری بر کاهش نشانه های اختلال لجبازی و نافرمانی در کودکان 8 تا 11 سال

http://tbj.ssu.ac.ir/article-1-2514-fa.html

مقدمه: کودکان و نوجوانان مبتلا به اختلال لجبازی و نافرمانی (ODD) الگوی رفتار منفی، خصمانه و نافرمان نشان می دهند که به مشکلات خانوادگی یا تحصیلی مهمی منجر می شود.اغلب کودکان، مخصوصاً در نوجوانی، یک دوره منفی گرایی و نافرمانی خفیف را پشت سر می گذارند و اغلب والدین از خصومت یا جر و بحث کردن های گاه و بی گاه فرزندان شان شاکی هستند؛ بنابراین هدف پژوهش حاضر بررسی اثربخشی بازی درمانی بر علائم اختلال لجبازی نافرمانی کودکان می باشد. روش بررسی: روش پژوهش از نوع نیمه تجربی است. در این مطالعه 15 نفر در گروه آزمون (7 کودک دختر و 8 کودک پسر ) و 15 نفر در گروه کنترل (9 کودک پسر و 6 کودک دختر ) قرار گرفتند. ابزار اندازه گیری شامل سیاهه رفتاری کودک CBCL ، فرم گزارش معلم TRF و یک مصاحبه بالینی با والدین بود. یافته ها: نتایج تحلیل نشان داد که علائم اختلال لجبازی نافرمانی به گزارش والد و به گزارش معلم کاهش معنی داری داشت. بنابراین بازی درمانی با رویکرد شناختی درمانی ، میزان نشانه های اختلال نافرمانی مقابله ای در کودکان پسر و دختر را در مرحله پس آزمون و پیگیری به طور معنی داری کاهش داده است . بحث و نتیجه گیری: این یافته ها اثربخشی بازی درمانی گروهی را برای کاهش علائم کودکان دارای اختلال لجبازی نافرمانی نشان می دهد و بر این اساس استفاده از این روش درمانی در مورد این کودکان به درمانگران توصیه می شود.

Title: بررسی موارد استفاده از بافت‌های غیرمجاز در همبرگرهای دست ساز در شهر تهران

http://tbj.ssu.ac.ir/article-1-1527-fa.html

مقدمه: امروزه فرآورده‏های گوشتی آماده پخت و مصرف بسیار مورد توجه مصرف کنندگان بوده و همبرگر بعنوان یک فراورده‌های گوشتی خام منجمد که تولید آن اغلب به صورت صنعتی است، از آن جمله می باشد. اخیرا تولید همبرگرهای دست ساز در صنف با مجوز معاونت بهداشتی وزارت بهداشت صورت پذیرفته و جایگزین‌های گوشتی غیرمجاز نظیر آلایش نامطلوب حیوانات کشتاری (ریه، پستان، بافت‌های طحال، اندام‌های داخل حفره شکمی، سنگدان مرغ، پوست و غیره) در فرمول ترکیبی، از تقلبات موجود در این فراورده می‏باشد. هدف این مطالعه تعیین موارد استفاده از بافت های غیر مجاز در همبرگرهای دست ساز عرضه شده در شهر تهران به روش‌بافت‏‌شناسی بوده است. روش بررسی: در این مطالعه در سال 1393، پس از تعریف مطالعه به صورت توصیفی، لیست اغذیه فروشی‌ها در مناطق مورد نظر شهر تهران تهیه و ضمن مراجعه حضوری بصورت تصادفی ساده، تعداد 42 نمونه همبرگر موجود تولید شده به روش دست ساز نمونه برداری و پس از ارسال به آزمایشگاه با حفظ شرایط سرد، از نظر وجود بافت‏های غیرمجاز و جایگزین‏های غیرمجاز گوشت در فرمولاسیون به روش بافت شناسی (رنگ آمیزی با هماتوکسیلین و ائوزین) مورد آزمون و بررسی قرار گرفت. یافته ها: در این مطالعه، میزان درصد موارد استفاده از بافت‏های غیرمجاز در نمونه‌های دست‌ساز 76/54 % شامل خمیرمرغ (19مورد) و پوست (4 مورد) مشاهده شد. آنالیز آماری داده‏ها نشان داد که استفاده از خمیر مرغ به عنوان تقلب در تولید همبرگر دست ساز اختلاف معنا داری در مقایسه با استفاده از پوست دارد (05/0P<). نتیجه گیری: باتوجه به اینکه استفاده از بافت‏های غیرمجاز در همبرگر براساس استانداردهای موجود، تقلب محسوب می‏شود و سلامتی مصرف‏کنندگان را به مخاطره می‏اندازد، لذا لزوم کنترل‌ و نظارت‌های دقیق بهداشتی بر اغذیه‌ فروشی‌ها ضروری است.

Title: تاثیر آموزش مبتنی بر مدل اعتقاد بهداشتی HBM) ( بر عملکرد بهورزان در نمونه گیری التور از مراجعین به خانه های بهداشت 1395

http://tbj.ssu.ac.ir/article-1-2237-fa.html

مقدمه : نمونه گیری التور از مراجعین به خانه های بهداشت عملکردی است که انجام صحیح و مرتب آن توسط بهورزان تأثیر قابل توجهی در تآمین و حفظ سلامت روستائیان و به تبع آن در شهرها ، دارد لیکن وضعیت انجام این رفتار تا به حال در تحقیقات مورد توجه قرار نگرفته است لذا این مطالعه با هدف تعیین میزان تاثیر برنامه آموزشی مبتنی بر مدل اعتقاد بهداشتی بر عملکرد بهورزان در نمونه گیری التور از مراجعین مبتلا به اسهال در سطح خانه های بهداشت شهرستان تفت در سال 1395-1394 انجام شد. روش بررسی:  این پژوهش مداخله ای از نوع تجربی و نمونه آماری 90 نفر شامل 52 زن و 38 مرد بودند که به صورت سرشماری از کلیه بهورزان شهرستان تفت در تحقیق وارد شدندو به طور تصادفی در دو گروه آزمون و شاهد قرار گرفتند. تجزیه و تحلیل  داده ها توسط نرم‌افزار آماری SPSS   و با استفاده  از آمار توصیفی مثل میانگین، انحراف معیار ، جداول توزیع فراوانی و هم چنین از آمار استنباطی نظیر  آزمون های ‌ T-testمستقل و زوجی انجام شد . در این آزمون سطح معنی داری آزمون 05/0 در نطر گرفته شد . یافته‌ها: نتایج حاصل از مداخله آموزشی نشان داد که قبل از اجرای مداخله آموزشی اختلاف آماری معنی داری بین دو گروه آزمون و شاهد از نظر سازه های مدل اعتقاد بهداشتی وجود نداشت. در گروه آزمون اختلاف میانگین نمرات رفتار نمونه گیری التور ، آگاهی ، حساسیت درک شده ، شدت درک شده ، منافع درک شده ، موانع درک شده، راهنما برای عمل و خودکارآمدی قبل و دو ماه بعد از مداخله از نظر آماری معنی دار بود به گونه ای که بعد از اجرای مداخله میانگین نمرات سازه های مدل اعتقاد بهداشتی افزایش یافته بود و سازه موانع درک شده کاهش معنی داری داشت  ( 001/0p< همه سازه ها ).  نتیجه گیری : بر اساس نتایج مطالعه، مداخله آموزشی بر مبنای الگوی اعتقاد بهداشتی می‌تواند سبب ارتقاء رفتار نمونه گیری التور از مراجعین مشکوک به وبا و بهبود سازه های الگو در بهورزان درسطح       خانه های بهداشت گردد .

Title: بازدارندگی فلزات سنگین و حلال‌ها بر آنزیم اوره‌آز برای تولید آمونیاک در سنتز داروی ضدفشار خون نیفیدیپین

http://tbj.ssu.ac.ir/article-1-2565-fa.html

مقدمه: رشد تکنولوژی‌ با مخاطرات زیست‌ محیطی بوسیله حلال‌های آلی و کاتالیست‌های سنتی همراه شده و لذا این مواد باید جای خود را به آب و بیوکاتالیست‌های زیست‌ سازگار بدهند. اوره‌آز، یک آنزیم هیدرولاز است که اوره را به آمونیاک تبدیل می‌کند، پس مخلوط اوره‌آز-اوره در آب یک جایگزین مناسب برای ماده سمی آمونیاک در سنتز نیفیدیپین است که در حضور کاتالیست‌ها و حلال‌های آلی زیادی سنتز شده است. هدف این تحقیق، جایگزینی اوره‌آز-اوره و آب به جای آمونیاک و مطالعه بازدارندگی مهار‌کننده‌های فلزات سنگین و حلال‌ها روی آنزیم اوره‌آز برای تولید آمونیاک در سنتز داروی ضد‌فشار خون نیفیدیپین است. روش بررسی: در این روش کاملاً تجربی، با کنترل بازده جداسازی/زمان واکنش سنتز نیفیدیپین از تراکم چهار جزئی متیل‌استواستات، 2-نیتروبنزالدهید و اوره‌آز-اوره در آب، شرایط بهینه مشخص و سپس با تغییر در فاکتورهای غلظت آنزیم، اوره، دما، pH و بازدارنده‌های فلزات سنگین/حلال‌های آلی، اثرات آنها روی سنتز نیفیدیپین کاتالیست شده با اوره‌آز بررسی ‌شد. یافته‌ها: بر اساس بازده نیفیدیپین، فعالیت آنزیم اوره‌آز برای تولید آمونیاک به عواملی چون مقدار اوره-اوره‌آز، دما، pH، نوع و غلظت بازدارنده‌ بستگی دارد. اوره‌آز به‌طور ویژه سوبسترای اوره را با ماکزیمم فعالیت در شرایط mg/mL 10 اوره‌آز، 7=pH و دمای C° 70 به آمونیاک تبدیل‌می‌کند و یون‌های فلزات سنگین و حلال‌های آلی با روند بازدارندگی Hg2+>Ag+>Cu2+>Pb2+>Cd2+ و استونیتریل>کلرو‌فرم>دی‌کلرو‌متان باعث کاهش فعالیت اوره‌آز و بازده نیفیدیپین می‌شوند. نتیجه‌گیری: اگر چه اوره‌‌آز-اوره یک منبع زیست‌سازگار آمونیاک در سنتز داروی ضدفشار خون نیفیدیپین در آب است، عوامل بازدارنده آنزیم اوره‌آز مانند یون‌های فلزات سنگین به ویژه جیوه و حلال‌های آلی باعث توقف این واکنش می‌شوند. زیست‌سازگاری کامل روش و کاتالیست، استفاده از حلال آب، انتخاب‌گری و بازده بالا، از مزایای این روش در مقایسه با روش‌های قبلی ‌است.
